# Airway management: How current are we?

**DOI:** 10.4103/0019-5049.76565

**Published:** 2011

**Authors:** Venkateswaran Ramkumar

**Affiliations:** Chairman, All India Difficult Airway Association (AIDiAA), Professor and Head of Anaesthesiology, Kasturba Medical College, Manipal - 576 104, India. E-mail: rvenkateswaran@gmail.com

Maintaining a patent airway is the primary responsibility of every anaesthesiologist and intensivist. It is a prerequisite for adequate gas exchange. Though airway related problems can be encountered in any subspeciality of anaesthesiology, it may be more commonly seen in certain surgical disciplines such as head and neck, cervical spine, trauma, bariatric, obstetric, paediatric and neurosurgery. Anticipated airway problems can be managed safely with the help of a protocolised, well-practiced algorithm that guides the anaesthesiologist through the logical steps of airway management. An unanticipated difficult airway often strikes like a bolt from the blue and may result in an adverse outcome if the concerned anaesthesiologist is either not abreast with current guidelines and/or is not familiar with the use of a variety of airway adjuncts that are currently available.

Failure to maintain a patent airway for more than a few minutes results in brain damage and even death. The Closed Claims Analysis from USA revealed that more than 85% of respiratory-event related closed malpractice claims involved a brain-damaged or a dead patient.[1,2] Problems related to airway management at the time of intubation as well as during emergence from anaesthesia remain among the leading causes of serious intraoperative problems.[3]

The standards of anaesthetic care in the Indian scenario are as diverse as the country itself, and vary even within the same city depending on whether one is looking at a small hospital practice or an institutional practice. This is not unexpected as there are no clear Indian guidelines that spell out what minimum equipment should be available and what course of action should be taken when faced with an anticipated or an unanticipated difficult airway during elective or emergency surgery. International guidelines from the United States of America and the United Kingdom provide a framework for structured decision making when faced with a difficult airway.[4,5] These algorithms provide a suggested plan of action and do not constitute a mandatory code of practice even in the country of origin. As these algorithms are country-specific, they cannot be directly applied to the Indian scenario.

There has always been a pressing need to determine the current Indian scenario with reference to the management of the difficult airway. With this in mind, the author designed a *Questionnaire* that was sent out by e-mail to around 600 anaesthesiologists. The consensus of opinion of the anaesthesiologists who responded is summarised below, tempered with the (hopefully unbiased!) interpretation of the author.

## DEMOGRAPHY OF RESPONDERS AND AWARENESS OF AIRWAY MANAGEMENT PROTOCOLS

The majority of responders (70%) were young anaesthesiologists who had been in anaesthetic practice for a period of less than 10 years (with approximately half of them having been in practice for less than 5 years). Thirty per cent of the responders were practitioners who had more than 10 years of professional standing. The majority of responders (80%) encountered less than 5 patients with difficult airway every month, while the remaining 20% faced the situation more frequently. Paediatric difficult airways were commonly encountered by the cross section of anaesthesiologists who responded (four of every five responders). Thus, the problem of a difficult airway is certainly there and one is likely to encounter it sooner than later.

It was heartening to note that 90% of the Indian responders were aware of difficult airway management protocols such as the one outlined by the American Society of Anesthesiologists or the Difficult Airway Society (UK).[4,5] However, despite such a high degree of awareness of the presence of difficult airway algorithms, only 60% of the responders stated that they had a local version of a difficult airway management protocol in place in their own operating rooms. Though 9 out of 10 anaesthesiologists were aware of the presence of international guidelines for the management of a difficult airway, only 6 out of 10 actually had a formal, practiced difficult airway management protocol in place in their work environment. This situation is akin to an adventure lover who knows what to carry when he sets out on a trek through difficult terrain but does so without arming himself with appropriate maps and special trekking gear. Does the Indian scenario call for more proactive behaviour on our part? I believe that unbiased introspection is certainly the need of the hour in this important area to make our practice of anaesthesiology and intensive care more scientific and safe.

## AVAILABILITY OF A “DIFFICULT AIRWAY CART” AT ONE’S WORKPLACE

With the plethora of devices now available for the management of difficult airway, it is necessary that all airway equipment be available in an accessible place so that they can be obtained without delay when needed. Conceptually, a difficult airway cart provides this option. It should carry the entire range of airway devices and adjuncts consisting of simple oropharyngeal/nasopharyngeal airways, supraglottic devices, laryngoscope blades/handles, rigid optical laryngoscopes, flexible fibreoptic laryngoscopes and an emergency cricothyrotomy set.[6] A difficult airway cart makes the management of a difficult airway more efficient as all the necessary equipment to deal with most clinical situations are readily available in one place. The questionnaire revealed that a difficult airway cart was available at the workplace of 6 of every 10 responders. The simple format of the questionnaire did not permit evaluation of the actual components making up these carts though, in retrospect, such an opinion would have given better insight into what was actually available to the average anaesthesiologist practicing in the Indian context.

## CHOICE OF EQUIPMENT WHEN FACED WITH A DIFFICULT AIRWAY

The questionnaire aimed to assess the preference of anaesthesiologists regarding their choice of airway adjuncts or specialised airway equipment when faced with a difficult airway. The details of difficult airway scenarios were not spelt out in the questionnaire. Opinions expressed by the responders regarding the choice of airway equipment have been summed up below.

### Adjuncts to facilitate conventional laryngoscopy and intubation

When faced with a (can ventilate) cannot intubate situation, international guidelines suggest that three steps be followed after first calling for help. These include changing the position of the patient, changing the laryngoscope blade/handle and changing the laryngoscopist. It was encouraging to note that practically all the anaesthesiologists who responded to the questionnaire were of the opinion that obtaining an alternate laryngoscope blade or handle was an appropriate path to be followed in this situation. Nine of 10 responders also opined that they would consider the use of an intubating stylet or tube exchanger when faced with this situation. Both these steps are in absolute agreement with the current international guidelines.

The majority of responders (close to 80%) opined that the lighted stylet was not a device that they would reach out for at this juncture. Unfamiliarity with the equipment as well as its limited availability could be two possible reasons for this response. The lighted stylet has immense advantage in facilitating intubation in patients in whom cervical spine flexion and/or atlanto-occipital extension are either restricted or not permitted due to an unstable cervical spine. Even in the presence of a normal cervical spine, the lighted stylet can still be used in conjunction with conventional laryngoscopy to facilitate endotracheal intubation when the grade of laryngoscopy is poor. In this situation, the lighted stylet literally “throws light” into the trachea (seen as a midline “glow” in front of the neck) and leads the intubator into the trachea (“lead kindly light”).

### Supraglottic airways

Since its first description by Dr. Archie Brain in 1983, the laryngeal mask airway (LMA) has become the device of choice for oxygenating a patient when endotracheal intubation has failed.[7] It is very important to remember the old adage, “patients die because of failure to oxygenate; not because of failure to intubate”.

The LMA Classic is an excellent device that can be placed relatively easily to provide a means of oxygenating a patient. It is a device that can be used in the emergency room or even in the field situation as it does not need any ancillary equipment for clinical use. The intubating laryngeal mask airway (ILMA) represents a major advance over the LMA Classic as, in addition to providing a means of oxygenating the patient, it also creates a conduit through which the patient can be intubated using a special silicone endotracheal tube that comes with the device. The ProSeal LMA has a specially designed cuff that ensures a snug fit over the laryngeal inlet. It has a gastric drainage channel that approximates to the cricopharynx and facilitates the drainage of gastric contents away from the laryngeal inlet. The ProSeal LMA might provide a distinct advantage when faced with a “can’t intubate” situation during anaesthetic induction in a patient with a “full stomach”. The gastric channel also facilitates the passage of a Ryle’s tube for emptying the stomach. The C-Trach LMA, which is a further refinement over the ILMA, allows the user to perform the intubation under visual guidance using a monitor screen that is an integral part of the device.

The questionnaire revealed that 9 out of 10 responders chose to use the LMA Classic when faced with a difficult airway. This is testimony to the ease with which operators can learn to place this device following a rapid learning curve (of possibly 25 insertions). It also reflects the ubiquitous presence of this device in most operating rooms and emergency carts as it is relatively inexpensive compared to other difficult airway devices.

The ILMA and ProSeal LMA were chosen by 6 responders out of 10 as airway adjuncts for dealing with a difficult airway. Only 1 out of 10 responders chose the C-Trach LMA as a device they would consider in the difficult airway situation. Could unfamiliarity with the equipment, fear of the unknown and cost have resulted in the remaining responders not opting for these devices? Recent literature is replete with evidence supporting the use of these three members of the LMA family, namely the ILMA, ProSeal LMA and C-Trach LMA, in specific difficult airway scenarios. It is high time these devices become more easily available at an affordable price in India so that they can be used in preference to other devices when faced with a difficulty to oxygenate patients.

### Indirect laryngoscopes

Several devices are available that make use of a rigid or a flexible optical conducting system to “look around corners” when direct laryngoscopy fails to reveal a laryngoscopic view that is better than grade 3b or 4. All these devices have an added advantage as they can be used to facilitate intubation when movement of the cervical spine is either restricted (rheumatoid arthritis) or is not permitted (unstable cervical spine).

The Bullard laryngoscope is one such device that can be used when cervical spine pathology restricts flexion of cervical spine and/or extension at the atlanto-occipital joint. Like the lighted stylet, it also proves useful when mouth opening is restricted to just a few millimeters. Prohibitive cost and limited availability probably resulted in the majority of responders not listing the Bullard laryngoscope as a choice when faced with a difficult airway.

The AirTraq is a device that could be distantly likened to the Bullard laryngoscope in that it provides an indirect view of the larynx through an optical viewing system (either direct or through a wireless remote viewing screen). This system facilitates passage of the tube “under vision”. The Glidescope is again a similar device that has an optical viewing system at the tip of a conventional laryngoscope blade that gets transmitted to the viewing screen through a cable. Both these devices were not popular in a majority of responders. Unfamiliarity with the equipment and prohibitive cost could be the only plausible reasons for this response.

The flexible fibreoptic laryngoscope is arguably the most versatile of the indirect laryngoscopes designed to facilitate intubation. The biggest advantage of this device is its “flexibility”, a feature that gives the operator the ability to use it nasally even in situations where there is absolutely no mouth opening. It can also be used safely and atraumatically to assess the upper airway with minimal discomfort to the patient prior to formulating an airway management plan for a difficult airway. Prohibitive cost is the only disadvantage of this device. But on a philosophical note, can one place a price on human life? The answer is an obvious and emphatic “NO”!! It is therefore no wonder that three of every four responders chose the flexible fibreoptic laryngoscope as an option when faced with a difficult airway. The flexible fibreoptic laryngoscope is probably more popular than the optical laryngoscopes as it has been in the market for long enough to be considered as the “gold standard” in airway management.

### Other techniques/surgical airway

Before modern airway adjuncts or specialised airway equipment became available in the market, most anaesthesiologists depended on techniques such as blind nasotracheal intubation, retrograde intubation and cricothyrotomy to deal with a difficult airway. How many modern anaesthesiologists are competent or confident in performing these relatively more invasive techniques? Two of 3 responders felt they were competent to perform blind nasotracheal intubation. One of every 4 declared competence to perform retrograde intubation while half the responders stated competency in performing cricothyrotomy. These facts are important as these relatively more invasive techniques may still work when modern equipment have failed to secure the airway. Training in the performance of these invasive lifesaving techniques needs to be adequately emphasised during the conduct of hands-on difficult airway workshops.

## AWARENESS OF MORBIDITY AND MORTALITY ASSOCIATED WITH “CAN’T VENTILATE, CAN’T INTUBATE” SITUATIONS

Seven of 10 responders were aware of morbidity or mortality related to “can’t ventilate, can’t intubate” situations in their institution. In retrospect, it would have been interesting to follow up those responders who had answered in the affirmative. Had an audit been done to identify the actual reasons for morbidity and mortality? Had any corrective action been taken in the form of procuring new equipment? Had the concerned personnel been given the opportunity to update their knowledge or had appropriate skill training been provided? Had the lesson learnt from this experience been adequately internalised? These are the questions that one needs to ponder over in an honest and unbiased manner if one is keen on improving safety issues relating to the way in which we practice anaesthesiology.

## TRAINING IN DIFFICULT AIRWAY MANAGEMENT

Only 4 of every 10 responders had actually attended a workshop on difficult airway management techniques in the previous year. Does this reflect a paucity of such workshops being held at the regional level? Or does it indicate a lack of interest in attending such workshops? The questionnaire provides the answer to the second question. It was encouraging to note that 95% of the responders would consider registering for a workshop dealing with difficult airway management techniques if one were to be held in their state or zone. This indicates the positive mindset of most anaesthesiologists who responded to the questionnaire. The majority indicated a definite interest in improving their knowledge and skills in this important area of anaesthetic care.

## DIFFICULT AIRWAY MANAGEMENT: THE FUTURE

It is evident that there is a need for updating our knowledge and skills in airway management. Though workshops that specifically address these issues are being held, they are few and far apart. The formation of the All India Difficult Airway Association (AIDiAA) (www.aidiaa.org) augurs well for those amongst us with interest in airway management. The manifesto of the AIDiAA is to first identify the training needs amongst anaesthesia professionals (qualified as well as those who are under training) and then to make such training opportunities available at their doorstep. It is also time for anaesthesia educators in charge of postgraduate training programmes to ensure that their postgraduates have access to a well-designed difficult airway rotation. This multipronged approach will hopefully address all the present problems in this all important area of anaesthetic management. The future generation of anaesthesiologists will surely be better airway managers. Though we have a long way to go, it is my firm belief that we have taken the first step in the right direction. The future of airway management does look promising!
